# Supplementary material for: Word synonym relationships for text analysis: A graph-based approach
Source: PLoS One. 2021 Jul 27;16(7):e0255127. doi: 10.1371/journal.pone.0255127 (PMC8315826; doi:10.1371/journal.pone.0255127)
Supplement: S1 Text — Twitter handles were anonymized. (PDF) [file pone.0255127.s002.pdf]

S1 Text.
The replies associated with the tweet in the Illustrative example Section. Twitter handles were anonymized.

1. When I fast in Ramadan like billions of Muslims, the month ends and I am healthier, my skin is better and loosing weight.

2. That's what Ramadan does when you fast. When you not eat for one month for several hours a day. You purify your body, your soul, while connecting with the Superior Being. Science now slowly proving what #ProphetofCompassion told us 1400 years ago.

3. this concept has been around since ancient times.. way before islam was created

4. I get grouchy if I haven't eaten, so bite me.

5. Why not stop eating altogether. Solve so many problems at once.

6. WTF? My whole life I've heard that eating 5 6 small meals throughout yoir day is better than 3 ”large” ones. And that ”grazing” speeds up your metabolism. While ”starving yourself” slows it down and cause the body to horde fat. So, which is it? Rhetorical. It's the former.

7. who told you you gotta eat three times or even more a day and who told you you gotta eat each time till you filled up complete day after day

8. And possibly develop stomach ulcers

9. Intermittent fasting is great. Easy way to lose weight

10. It actually works, I lost 19 kg this way and the healthiest I have ever been. Food no longer control my life. And also extended fasting was what cured my allergies. So win-win for me also save lots of money since I normally eat one meal a day, sometimes two.

11. But i have that murdery mood

12. It's Muslim http://fasting.It's compulsory for a lunar month,optional twice a week.Other times fasting is a preferred way of teaching oneself control,discipline & piety. Foodstuff production & engineering 2 meet demand based on materialism& indulgence is culprit of health ills.

13. Stop eating at 5pm - have breakfast between 9am- 11am. That's intermittent fasting. Harder than it sounds though
14. That's what Ramadan is all about.
15. yeah people crazy eating whole day long
16. The last thing I want is to live longer
17. Intermittent fasting isn't hard if you're eating the right things. I only eat two meals a day and fast for 16 hours and I'm never hungry. I lost 30lbs doing it along with clean Keto.
18. @XX 16-18 hours??? I doubt its ever been more than 30 minutes since I've ate
19. In simpler words: Ramadan Fasting But will it hurt since it's more of a Muslim thing??
20. @XX Wow!
21. Remember a time when Western people thought Muslims were following obsolete way of life by doing this same thing.
22. America please jump on board with fasting. I'm so tired of being from a country that is most known for its obese inhabitants!
23. Majority of the 1.6 billion Muslims does this for 30 days every year during Ramadan. Something our holy prophet Mohammed (peace be upon him) taught us 1400 years ago
24. If I don't eat a dead animal 3 times a day, it doesn't count.
25. Breakfast is a lie
26. Most people eat to live; others live to eat. Moderation is best.
27. Or doing this! Watch
28. Um, how about NO.
29. Doubt it
30. Hes at it again bor.
31. So that means that the poor and the homeless will inherit the earth?
32. @XX Here we go again! Thank you for the great mental, physical, healthy, psychological and masculinity guidelines.
33. Yes been doing this since i was a kid. Feel so good after Ramadan. Its the best
34. Better late than never
35. Or dairy and coffee and red wine could be good for you. Let's wait for the next study to find out for sure.
36. Lent that lasts 40days is my time to fast. It's a real trial but spiritually and physically refreshing
37. The article doesn't mention that fasting allows one to drink water.

38. Let demosatans Congress go 24/7 and test it out.
39. It's not hunger, it's your body being used to food all times of day. Grelin.
40. Irresponsible post. Need to preface this with excluding women. A lot of women. Don't do well with even a 12 hour fast.
41. I went from 285+ to 165 at the age of 50 (spent 20 yrs weighing 240 - 290 or so) and have kept all the weight off for over a year now. I fast for 20+ hrs and will never go back to a "regular" eating schedule.
42. Opens box of oreos* im here for a good time not a long time.
43. But I may kill a couple people from being hungry
44. I don't even want to live another day let along any longer! Thanks to fake news!
45. Ramadan and the voluntary fasting
46. RAMADAN BISHES!!! We ARE the chosen ones.
47. So Ramadan?
48. "Sounds like Ramadan and the routine practice of Prophet Muhammad (pbuh) " said Muslims everywhere
49. *Laughs/Dances in Muslim*
50. Alhamdullah
51. You wanna stop repetitively posting pro-anorexia stuff please thanks
52. Why live longer if it's just a miserable existence? I've been poor and hungry, it was...unpleasant. I'm not about to purposely starve myself.
53. Muslims doing it for over 14000 years once a month every year.
54. I'm sure your blood pressure and blood sugar will drop and you'll lose weight if you're basically starving yourself. Bad take @ whatever scientist came up with this
55. Eat one good meal of food a day for good health. One meal a day or every two days could spell the end of much medicine, doctor bills and the building of hospitals if we would eat the proper food and eat it only when it is necessary to eat it.
56. Naaah when I am hungry I just tuck in. The yoga stuff is not for regular folk. Good day snowflakes!
57. "The clinical trials focused on overweight young and middle-age adults, so the benefits and safety can't be generalized to other groups, the authors said." That's kind of important. If your longevity is decreased due to obesity, wouldn't *any* healthier change be positive?
58. I once had a boss that said eating was just a habit...
59. So anorexia works? Groundbreaking.
60. Laughs in Muslim
61. More Asians have heart attacks earlier than most western nations !

62. Muslims be like
63. The adverse effects might not be worth it in the long run. Something healthy and that doesn't restrict how much you eat? A plant based diet.
64. Doing it
65. Elderly fall when they don't eat. They need to graze since they never KNOW which time their body fully absords nutrients. You can stick that where the sun does not shine
66. It's been 8365 retweets for days now. You think it's about time CNN?
67. Only for obese people!!!
68. 'Pushing past the hunger' for many people is not a safe thing to do.
69. Why are people getting so upset about this? It's not more difficult than any other sort of diet, and has no mention of outright restriction of foods. Also it's a suggestion for addressing the very real health problems stemming from stress and obesity. There is nothing to offend.
70. *ahem* Same nonsense different year
71. Gross. Please don't glorify disorder disordered eating
72. Good grief, most people fast for at least 12-14 hours anyway. If they don't pig out before bed. Thinking this is some astonishing thing.. lol
73. A autistic friend of mine with diabetes-1 did extreme intermittent fasting.
74. Yea it's called burning calories- your stomach rumbles- not really a good thing. Get off your GD Arse and exercise
75. Everyone from dietitian, hospital and diabeticdocter said it was a bad idea His results were - perfect blood work - required 60% less insuline - lost 25kg weight So yes it works. But be careful!
76. now imagine you would use all the calories you're eating you would be twice as fat as you are the most you eat you drop out unused anyway I mean the people who are eatin all day long
77. Eat food. This shit is mentally unhealthy and can have negative affects on the body.
78. How many times you gonna tweet this shit?
79. ...bullshit!!
80. Trying to get people ready for socialism?
81. Oh you people with your plentiful food and options to eat and not eat FIRST WORLD PROBLEMS!
82. Abstaining from food for 18 hours a day hopefully you do not have an 8 hour a day job because you only have 6 hours a day to eat.
83. Don't knock it if you haven't tried it! It's the healthiest way to eat ever so give it a try before you say it's stupid or doesn't work.

84. I'm here for a good time, not a long time. What's everyone's obsession with trying to live long? Need extra time to complain about miserable they are, I assume.

85. EAT ONCE EVERY 24 OR ONCE EVERY 48 HOURS AND YOUR HARDLY EVER GET SICK!!! ONCE EVERY 72 HOURS AND BLOW SICKNESS OUT OF YOUR LIFE !!!

86. Let's be honest, Americans aren't capable of abstaining from food. Any other culture on the planet could do it, but the Americans can't even give up guns and you want them to give up food? They're overweight for a reason.

87. It's not at all difficult to stick to. Easiest diet of my life and I'm 15 pounds below my goal weight.
