# Supplementary material for: Word synonym relationships for text analysis: A graph-based approach
Source: PLoS One. 2021 Jul 27;16(7):e0255127. doi: 10.1371/journal.pone.0255127 (PMC8315826; doi:10.1371/journal.pone.0255127)
Supplement: S1 Appendix — (PDF) [file pone.0255127.s003.pdf]

S1 Appendix.

Community extraction using the Leiden algorithm. S1 Table shows the community partitions associated with the text graph discussed in the Illustrative Example Section (Fig 3) extracted using the Leiden communitiy detection algorithm. The set of keywords extracted from the Leiden communities is as follows.

> $kw = \{$work, sort, lot, effect, focus, mood, complete, dead, fat, best, well, safety, blood, problem, disorder, open, capable, earlier, base, world, group, minute, blow, attack, great, large, sick, nutrient, long, even, speed, word, muhammed$\}$.

A quick comparison between the communities extracted using the two algorithms: Louvain (Table 2) and Leiden (S1 Table) shows that the Leiden algorithm was able to extract fewer but stronger communities. However, a lower overall performance of the proposed method was observed.

S1 Table. Summary of the qualities of the 15 main communities in the text graph associated with the example in Section: An illustrative example.

No.	C_i (sorted by degree)	$weight(C_i)$	$\|V_{C_i}\|$	$\|E_{C_i}\|$	$size(C_i)$	$density(C_i)$	$diam(C_i)$	$CC(C_i)$	Quality
1	work, ready, shit, cause, building, practice, fake, exercise, sort, kind, use, habit, pig, test, plant, bullshit, lot, change, trial, year, mental, concept, doctor, people	48	24	94	118	0.09	5	0.5	High
2	effect, pressure, real, push, heart, way, focus, stuff, board, really, actually, whole, altogether, mood, stress, bill, result, age, life, weight	37	20	44	64	0.06	9	0.2	Low
3	complete, last, goal, back, end, live, stomach, dead, culture, bed, perfect, gross, address, pound, fat, spell, arse, post, extreme, everywhere, plentiful, engineering	32	22	76	98	0.08	5	0.4	Low
4	best, good, sound, right, safe, well, honest, healthier, healthy, healthiest, safety, proper, hardly, fully	30	14	86	100	0.2	2	0.9	High
5	blood, job, problem, grazing, disorder, fast	27	6	10	16	0.2	4	0	Low
6	open, loose, easy, discipline, clean, capable, late, study, watch, control, slowly, earlier, unused, former, option, mention, superior	20	17	68	85	0.1	6	0.5	High
7	base, earth, small, miserable, stem, world, country, reason, idea, nation, group, first, existence, poor, kid, hurt	19	16	42	58	0.1	5	0.6	Low
8	minute, bite, burning, blow, routine, attack, hour, gun	17	8	16	24	0.1	6	0.2	Low
9	great, hard, bad, harder, large, overweight, ill, adult, sick, difficult, crazy, adverse	16	12	48	60	0.2	4	0.7	High
10	nutrient, food	11	2	2	4	1	1	1	High
11	long, longer	10	2	2	4	1	1	1	High
12	even, regular	6	2	2	4	1	1	1	High
13	speed, nothing	2	2	2	4	1	1	1	High
14	word, news	2	2	2	4	1	1	1	High
15	muhammed, mohammed	2	2	2	4	1	1	1	High
