# Supplementary material for: Word synonym relationships for text analysis: A graph-based approach
Source: PLoS One. 2021 Jul 27;16(7):e0255127. doi: 10.1371/journal.pone.0255127 (PMC8315826; doi:10.1371/journal.pone.0255127)
Supplement: S2 Appendix — (PDF) [file pone.0255127.s004.pdf]

S2 Appendix.

Keywords using iterative community extraction. It can be observed from Table 2 that larger communities tend to exhibit lower qualities. The community quality of a community C_i can be enhanced by partitioning its vertices iteratively into smaller (stronger) communities. S1 Table shows the communities extracted from each low quality community with $|V_{C_i}| > 10$ in Table 2. S2 Table shows the communities extracted from each low quality community in S1 Table. The set of keywords kw extracted using the new communities is

kw = {discipline, watch, capable, exercise, use, practice, sort, good, blood, disorder, change, year, work, trial, spell, last, culture, live, complete, perfect, everywhere, arse, bed, back, post, pound, fat, engineering, building, concept, fake, shit, pig, people, lot, ready, doctor, plant, reason, weight, life, real, whole, bite, attack, burning, harder, food, long, loose, miserable, hurt, regular, ill, earth, stem, thank, speed, yea, ate, word, muhammed, ramadan, muslim, month, day, body, time, many, meal, intermittent, ever, also, twice, health, breakfast, thing, never, hungry, doubt, western, obese, something, prophet, yes, sure, hunger, woman, much, hospital, obesity, diet, anyway, calorie, everyone, american}.

S1 Table. Communities extracted from low quality communities in Table 2.

C_i	Vertices (sorted by degree)	$weight(C_i)$	$\|V_{C_i}\|$	$\|E_{C_i}\|$	$size(C_i)$	$density(C_i)$	$diam(C_i)$	$CC(C_i)$	Quality
■	■$_1$: discipline, control, watch, study, capable, superior	7	6	16	24	0.3	3	0.5	High
	■$_2$: exercise, habit, use, practice	5	4	12	16	0.5	1	1	High
	■$_3$: sort, kind, change, mental, year	11	5	10	15	0.25	3	0.3	Low
	■$_4$: work, test, trial, routine, spell, stress	11	6	14	20	0.2	3	0.6	High
■	■$_1$: last, end, culture, goal, live, extreme	13	6	18	24	0.3	2	0.8	High
	■$_2$: complete, dead, perfect, gross, everywhere	5	5	14	19	0.35	2	0.7	High
	■$_3$: back, arse, stomach, post, bed, pound	8	6	12	18	0.2	3	0.4	Low
	■$_4$: fat, plentiful	3	2	2	4	1	1	1	High
	■$_5$: engineering	1	1	0	1				High
■	■$_1$:building, concept	2	2	2	4	0.5	1	1	High
	■$_2$: fake, shit, pig	4	3	6	9	0.5	1	1	High
	■$_3$: lot, ready, plant, doctor, people	16	5	10	15	0.25	3	0.3	Low
	■$_4$: reason, cause	2	2	2	4	0.5	1	1	High
■	■$_1$: heart, effect, weight, life	13	4	6	10	0.25	3	0	Low
	■$_2$: real, actually, really, stuff	6	4	8	12	0.3	2	0.6	High
	■$_3$: whole	2	1	0	1				High

S2 Table. Communities extracted from low quality communities in S1 Table.

C_i	Vertices (sorted by degree)	$weight(C_i)$	$\|V_{C_i}\|$	$\|E_{C_i}\|$	$size(C_i)$	$density(C_i)$	$diam(C_i)$	$CC(C_i)$	Quality
■$_3$	■$_{31}$: sort, kind, change, year, mental	11	5	10	15	0.25	3	0.3	Low
■$_3$	■$_{31}$: arse, bed	2	2	2	4	0.5	1	0	High
	■$_{32}$: back, stomach, post, pound	6	4	8	12	0.3	2	0.6	High
■$_3$	■$_{31}$: people, lot	13	2	2	4	0.5	1	1	High
	■$_{32}$: ready, doctor, plant	3	3	4	7	0.3	2	0	Low
■$_1$	■$_{11}$: weight, effect	7	2	2	4	1	0.5	0	High
	■$_{12}$: life, heart	6	2	2	4	1	1	1	High
