# Supplementary material for: Word synonym relationships for text analysis: A graph-based approach
Source: PLoS One. 2021 Jul 27;16(7):e0255127. doi: 10.1371/journal.pone.0255127 (PMC8315826; doi:10.1371/journal.pone.0255127)
Supplement: Attachment — Submitted filename: response to reviewers.pdf [file pone.0255127.s006.pdf]

Point-by-point response to the editor and reviewers' comments and concerns.

Response to Editor Comments

We thank the Editor for reading the manuscript and providing his constructive comments. We have taken the comments into consideration to improve the quality of the manuscript. Please find below a point by point response to each comment.

Comment 1.1: It would be interesting to add to relevant sections of text (e.g. introduction, conclusion) the limitations of the method so the reader can be aware of potential problems and advantages of using other approaches such as embedding techniques.

Response 1.1: A brief discussion on the limitation of the proposed method was added to the Introduction Section (lines: 72-77) and to the Conclusion Section (lines: 686-694).

Response to Reviewer 2 Comments

We thank the reviewer for their reading of the manuscript and for their constructive comments. We have taken the comments into consideration to improve the quality of the manuscript. Please find below a point by point response to each comment.

Comment 2.1: I appreciate the authors' thoroughness in addressing the concerns raised in the prior version. If they continue this line of inquiry, I urge them to consider other approaches of community extraction (e.g., k-cliques) that allow for overlapping communities, as this could conceivably lead to a more naturalistic set of keywords. But that is for the next paper, not this one.

Response 2.1: A note about this was added to the Conclusion Section in the revised manuscript (lines: 697 and 698) as a possible future work for research in this direction.

Response to Journal Requirements

Comment 3.1: Please review your reference list to ensure that it is complete and correct. If you have cited papers that have been retracted, please include the rationale for doing so in the manuscript text, or remove these references and replace them with relevant current references. Any changes to the reference list should be mentioned in the rebuttal letter that accompanies your revised manuscript. If you need to cite a retracted article, indicate the article's retracted status in the References list and also include a citation and full reference for the retraction notice.

Response 3.1: The reference list entries have been reviewed. The format of the majority of references have been updated to match the reference style of the journal. Moreover, we have added the doi and link to references whenever possible in the References Section of the updated manuscript.
